# Cartilage Repair Surgery: Outcome Evaluation by Using Noninvasive Cartilage Biomarkers Based on Quantitative MRI Techniques?

**DOI:** 10.1155/2014/840170

**Published:** 2014-05-04

**Authors:** Pia M. Jungmann, Thomas Baum, Jan S. Bauer, Dimitrios C. Karampinos, Benjamin Erdle, Thomas M. Link, Xiaojuan Li, Siegfried Trattnig, Ernst J. Rummeny, Klaus Woertler, Goetz H. Welsch

**Affiliations:** ^1^Department of Radiology, Klinikum Rechts der Isar, Technische Universitaet Muenchen, Ismaninger Strasse 22, 81675 Munich, Germany; ^2^Department of Neuroradiology, Klinikum Rechts der Isar, Technische Universitaet Muenchen, Ismaninger Strasse 22, 81675 Munich, Germany; ^3^Department of Orthopaedic and Trauma Surgery, University Medical Center, Albert-Ludwigs Universitaet Freiburg, Hugstetter Strasse 55, 79106 Freiburg, Germany; ^4^Musculoskeletal and Quantitative Imaging Research Group, Department of Radiology and Biomedical Imaging, University of California San Francisco, 185 Berry Street, Suite 350, San Francisco, CA 94107, USA; ^5^MR Center, Department of Radiology, Medical University of Vienna, Lazarettgasse 14, 1090 Vienna, Austria; ^6^Department of Trauma Surgery, Friedrich-Alexander-Universitaet Erlangen-Nuernberg, Krankenhausstrasse 12, 91054 Erlangen, Germany

## Abstract

*Background*. New quantitative magnetic resonance imaging (MRI) techniques are increasingly applied as outcome measures after cartilage repair. *Objective*. To review the current literature on the use of quantitative MRI biomarkers for evaluation of cartilage repair at the knee and ankle. *Methods*. Using PubMed literature research, studies on biochemical, quantitative MR imaging of cartilage repair were identified and reviewed. *Results*. Quantitative MR biomarkers detect early degeneration of articular cartilage, mainly represented by an increasing water content, collagen disruption, and proteoglycan loss. Recently, feasibility of biochemical MR imaging of cartilage repair tissue and surrounding cartilage was demonstrated. Ultrastructural properties of the tissue after different repair procedures resulted in differences in imaging characteristics. T2 mapping, T1rho mapping, delayed gadolinium-enhanced MRI of cartilage (dGEMRIC), and diffusion weighted imaging (DWI) are applicable on most clinical 1.5 T and 3 T MR scanners. Currently, a standard of reference is difficult to define and knowledge is limited concerning correlation of clinical and MR findings. The lack of histological correlations complicates the identification of the exact tissue composition. *Conclusions*. A multimodal approach combining several quantitative MRI techniques in addition to morphological and clinical evaluation might be promising. Further investigations are required to demonstrate the potential for outcome evaluation after cartilage repair.

## 1. Introduction

Quantitative magnetic resonance imaging (MRI) techniques are frequently applied as noninvasive biomarkers for detection of early articular cartilage degeneration before morphological cartilage loss occurs [1–5]. Hyaline cartilage consists of chondrocytes to about 1%; the extracellular matrix is composed of collagen (15–20%), mainly collagen type II, proteoglycan (PG; 3–10%), consisting of protein and glycosaminoglycan (GAG) side chains, and water (about 80%) [6, 7]. The combination of all components provides important viscoelastic properties, which are critical for a proper cartilage function [7, 8]. Early cartilage degeneration is characterized by collagen disruption and GAG loss, resulting in an increasing bulk water content and mobility [6, 9].

Correlations of quantitative MR biomarkers with histological and biochemical properties of cartilage were previously described [10, 11]. Whereas T2 relaxation time measurements are mainly sensitive to collagen disruption and water content [12–18], other techniques exist that are sensitive to GAG concentrations. GAG is largely responsible for the negative fixed-charge density of articular cartilage. Delayed gadolinium-enhanced MRI of cartilage (dGEMRIC) [19–21] and ^23^Na MRI (sodium MRI) [22, 23] reflect this fixed-charge density and consecutively indirectly measure GAG concentrations [24]. GAG chemical exchange saturation transfer (gagCEST) measures GAG concentrations by using the GAG molecules as endogenous contrast agents [25–28]. T1rho relaxation time measurements can investigate the slow-motion interactions between the macromolecule protons and bulk water protons and therefore indirectly measure GAG concentrations [29, 30]. Although T2 and T1rho preferentially correlate with different cartilage parameters, high correlations were found between these two parameters [31]. Further techniques are diffusion weighted imaging (DWI) [32] and diffusion tensor imaging (DTI) [33], which are sensitive to water mobility. Additional techniques have been described to correlate with cartilage degeneration. A zonal organization of normal articular cartilage is known, which may be detected by quantitative imaging techniques in laminar analyses [34, 35].

If cartilage defects have occurred, focal defects may be treated by different surgical cartilage repair techniques, including microfracture (Mfx), osteochondral autograft transplantations (OAT), and autologous chondrocyte implantation (ACI) or matrix associated ACI (MACI), respectively [36–39]. These techniques are known to result in different histological types of cartilage repair tissue [40–43]. Prediction of long-term outcome is essential for the individual patient as well as for individual therapy optimization. Therefore, this is a field of research with a strong translational interest by surgeons, radiologists, physicists, and experimental researchers. Besides clinical evaluation, magnetic resonance imaging (MRI) represents one of the most important diagnostic tools for postoperative followup. Morphological outcome evaluation may include scores such as the MOCART [44, 45] or the Henderson score [46–48]. Quantitative MR imaging may provide additional important information on subtle tissue changes that are morphologically not detectable. The intention of quantitative imaging is to assess cartilage repair tissue noninvasively and to provide highly sensitive outcome measurements without the need of an arthroscopy or even biopsy. It may potentially be used to predict and monitor cartilage repair maturation and outcome [14].

The present work specifically reviews the current literature on the use of quantitative MRI biomarkers for evaluation of cartilage repair that are applicable at clinical 1.5 T and 3 T MR scanners.

## 2. Materials and Methods

### 2.1. Identification of Literature

An electronic search in PubMed (http://www.ncbi.nlm.nih.gov/pubmed) was performed to identify relevant studies on biochemical MR imaging of cartilage repair for this review. Specific PubMed searches including the entry terms “cartilage repair imaging” (835 results) and “cartilage repair MRI” (729 results) as well as the specific terms “cartilage repair T2” (104 results), “cartilage repair T1rho” (11 results), “cartilage repair dGEMRIC” (27 results), “cartilage repair diffusion” (70 results), “cartilage repair DWI” (5 results), and “cartilage repair DTI” (1 result) were screened for relevant studies. Additional searches were performed for the the entry terms “microfracture imaging” (126 results), “microfracturing imaging” (13 results), “cartilage transplantation imaging” (733 results), “chondrocyte transplantation imaging” (291 results), “OATS imaging” (36 results), and “osteochondral transplantation imaging” (274 results) to exclude that relevant studies for each cartilage repair technique were missed by the main entry terms. Further searches were performed using the term “MRI” instead of “imaging”; it did not result in additional relevant references. Reference lists of previous important studies and reviews were screened [2–6, 14, 15, 26, 36, 37, 39, 46, 49–71]. Subsequently, additional searches with the same entry terms were performed in the databases “EMBASE” and “Cochrane Library.” However, these did not result in further, relevant results. Only studies in humans were included. References included the time span up to December, 2013, without starting date.

## 3. Results

### 3.1. Techniques

The quantitative MR imaging techniques T2 and T2* mapping, T1rho mapping, dGEMRIC, and diffusion imaging may be performed on most clinical 1.5 T or 3 T MRI scanners using product or research pulse sequences and standard hardware; most of the techniques are also possible at 7 T [72]. Sodium MRI can especially benefit from higher SNRs and gagCEST can especially benefit from higher spatial resolution when performed at 7 T [28, 51]. Sodium MRI requires special hardware and gagCEST has been only recently applied in cartilage, making these two techniques not broadly available for clinical use.

It is not entirely clear whether prolonged unloading during an MR examination influences quantitative values [73, 74]. Mamisch et al. stated that the difference in T2 values between the beginning and the end of the MR examination was more pronounced in cartilage repair tissue than in normal cartilage [75]. Therefore, standardization of the time of unloading is recommended when performing quantitative MR imaging.

#### 3.1.1. T2 and T2* Relaxation Time Measurements

Currently T2 relaxation time measurements are the most investigated noninvasive MRI biomarkers for evaluation of articular cartilage and cartilage repair tissue. Higher and more heterogeneous T2 values are thought to characterize collagen deterioration and increasing water contents [14, 76]. T2 measurements do not require contrast agent injections.

Different pulse sequences can be used for T2 mapping including spin echo (SE), multislice multiecho (MSME) SE, fast spin echo (FSE), and T2-prepared 3D spoiled gradient recalled (SPGR) sequences [49, 77]. Welsch et al. described T2 DESS sequences that provide the possibility to combine morphological and biochemical MRI in one fast 3D sequence [66]. T2 values still vary significantly between different acquisition methods and MR scanners [49, 66]. Further, magic angle effects may cause a prolongation of T2 at 55° through the anisotropy of collagen with respect to the main magnetic field [78]. SE or MSME sequences are most frequently used for T2 mapping. Long echo trains of SEs are acquired, while the numbers and values of echo times (TE) vary [6, 79]. In the osteoarthritis initiative (OAI), a MSME SE sequence with seven echoes (echo times of 10, 20, 30, 40, 50, 60, and 70 ms) is used [80]. A MSME experiment, excluding the first echo from the later fitting process, improves the T2 quantification, since this eliminates the effects from stimulated echo signal [16, 49].

In the following postprocessing procedure, the measured signal intensity at each echo time (TE) is fitted to a monoexponential decay function and measured pixel by pixel to calculate T2 values [6] (*S*(TE_*t*_) = *S*_0_ × exp⁡(−TE_*t*_/T2); *S* is signal intensity; *S*_0_ is equivalent to *S* at TE = 0). Weighted or nonweighted linear least squares or nonlinear least squares fitting methods are used [13]. Noise correction is recommended due to low signal-to-noise ratio (SNR) in the images acquired at the long TEs [13]. T2 relaxation time maps are generated from the calculated T2 values to perform further analysis.

The deep and calcified zones of articular cartilage are composed of dense collagen fibrils contributing to short T2 relaxation times. That is why standard (MSME) T2 mapping techniques may have limitations [81, 82]. In these deep cartilage areas, UTE T2* mapping may be more sensitive for tissue changes with T2 relaxation times of less than 10 ms [83]. T2* has shorter imaging times and the possibility of 3D acquisition and thereby providing greater spatial resolution [83]. In contrast to T2 mapping, T2* mapping uses a gradient echo (GE) pulse sequence and includes both T2 relaxation and coherent dephasing effects. T2* and T2 values are related by the equation 1/T2* = 1/T2 + *γ*Δ*B*_0_ (*γ* is gyromagnetic ratio of the observed nucleus; Δ*B*_0_ is magnetic field inhomogeneity). It is assumed that Δ*B*_0_ is only influenced by local magnetic susceptibility fields present at the cartilage-bone interface or within the cartilage microstructure. Despite not reflecting the exactly same ultrastructural components, T2 and T2* have been described to correlate with each other [49, 72, 83, 84]. However, the correlation coefficient was relatively low, especially for the deep cartilage layer.

#### 3.1.2. T1rho Relaxation Time Measurements

The time constant T1rho describes the spin-lattice relaxation time in the rotating frame by application of spin-lock (SL) techniques [7, 85–88]. T1rho values have been shown to increase with GAG (PG) content loss of the extracellular matrix of hyaline cartilage, with increases in bulk water and with cartilage softening, while being less dependent on collagen disruption [31, 85, 89–94]. T1rho relaxation time measurements do not require contrast agent injections.

Current T1rho quantification techniques use SE, FSE [89, 90] spiral imaging [31], echo planar imaging [95], or 3D GE sequences [93, 96]. The data can be acquired with 2D or 3D acquisitions. 3D techniques provide thinner slices and may be preferred due to the nonslice-selective nature of the SL pulse. In these techniques spins are tilted to the transversal plane by a 90° pulse and then locked in that plane by an SL pulse. The responsible long, continuous SL radiofrequency power pulse along B1 exceeds small local molecular magnetic fields. Therefore, no T2 or T1 relaxation but longitudinal T1rho relaxation along B1 takes place [7]. For readout purposes, another −90° pulse is applied that flips this spin-locked magnetization back to the *z*-axis. In the following series, different SL pulse durations (time of spin lock, TSL) are applied. Finally, T1rho may be determined by fitting the data to the T1rho decay curve, which is governed by an exponential equation (*S*(TSL) ∝ exp⁡(−TSL/T1rho)). Due to SAR limitations, usually a SL frequency ≤ 500 Hz is used. The TSLs applied for cartilage are between 0 and 80 ms. Noise correction is recommended due to low signal-to-noise ratio (SNR) in the images acquired at the long TSLs.

#### 3.1.3. Delayed Gadolinium-Enhanced MRI of Cartilage (dGEMRIC)

dGEMRIC is another quantitative cartilage MR imaging technique that correlates with the PG content of articular cartilage [2, 19, 97–101]. In comparison to T1rho, dGEMRIC is able to provide a direct measure of the GAG content. However, it requires the application of a negatively charged intravenous contrast agent, gadolinium diethylenetriamine pentaacetate anion (Gd-DTPA), that enhances T1 relaxation times [11, 19, 62, 98, 102–104]. The negatively charged Gd-DTPA molecule penetrates cartilage in an inverse relationship to the concentration of negatively charged GAG side chains of PG [24]. A depletion of GAG content in degenerated cartilage results in an accumulation of the paramagnetic gadolinium ions, following the principle of electroneutrality. This consequently accelerates T1 relaxation [105]. There are several different sequences for dGEMRIC available, depending on the MR scanner [72, 73, 106–111]. Data can be acquired using 2D or 3D techniques [102, 108, 112–114].

Usually, a double dose (0.2 mmol/kg) Gd-DTPA is applied [99]. About 45–120 minutes after contrast administration, postcontrast MRI is performed, although reaching maximum and equilibrium concentration may take longer [99, 115–117]. Additionally, an exercise period is required after contrast agent application, which influences the distribution of the contrast agent [118]. Usually, T1 relaxation time measurements precontrast (T1) and postcontrast application (T1-Gd) are used to determine the contrast agent concentration in cartilage [19, 105]. The delta relaxation rate is defined by 1/T1 − 1/T1-Gd [119]. The relaxation index (relative delta) is defined by the delta relaxation rate of repair tissue divided by the delta relaxation rate of normal hyaline cartilage [119]. While differences of native T1 dGEMRIC values (T1) in normal hyaline cartilage can be neglected, it remains a matter of current investigations whether determination of native T1 dGEMRIC values in precontrast MRIs is necessary for evaluation of cartilage repair tissue [110, 119–123]. Precontrast dGEMRIC values of repair tissue were increased in MACI repair tissue compared to normal cartilage at all time points after surgery [119]. Watanabe et al. correlated dGEMRIC after ACI with biopsies [119]. A significant correlation of relative delta relaxation rates with relative GAG concentration, but not with relative T1 relaxation times before (T1) or after contrast application (T1-Gd), was observed [119]. Other authors found high correlations and similar results for T1-Gd and delta relaxation rates after MACI and osteochondral transplantation, respectively [110, 124–126]. To date, most studies acquired pre- and postcontrast images and additionally reported delta values [110].

#### 3.1.4. Diffusion Weighted Imaging (DWI) and Diffusion Tensor Imaging (DTI)

Diffusion weighted imaging (DWI) can probe water mobility in articular cartilage [2, 32, 127, 128]. Water molecules diffuse in the space surrounding the extracellular matrix of the cartilage. In cartilage with an intact collagen network, water mobility is restricted. The increased mobility of water in a deteriorated extracellular matrix, representing early cartilage degeneration, can be assessed by DWI [129]. Therefore, by measuring the molecular movements of water within the cartilage tissue, DWI techniques can probe tissue microstructure changes. Using this technique, two equal paired magnetic field gradient pulses are applied with a time delay [39]. The two gradient pulses either have opposed polarity or have the same polarity with an interposed 180° radiofrequency pulse [39]. The paired gradient pulses, usually referred to as the diffusion gradients, cause dephasing of the protons that diffuse (move) during a given time delay. The acquired MR signal is related to the diffusion coefficient of the proton (Stejskal-Tanner equation) [39]. Stationary water protons lead to a high signal, moving protons within the time delay lead to a signal decrease. This experiment is repeated with varying diffusion gradient strength. For each acquisition, the overall diffusion weighting of the image is determined by a so-called *b*-factor [130]. In the following, an apparent diffusion coefficient (ADC) map is calculated from the images with different *b*-factors on a pixel-by-pixel basis [127] using the equation *S*(*b*) ∝ exp⁡(−b*ADC). ADC values were found to be elevated in degenerated cartilage [32, 131, 132]. Single-shot echo planar imaging- (EPI-) based diffusion sequences are the current gold standard of DWI but suffer from susceptibility artifacts and limitations in contrast. Alternatively, diffusion imaging can be performed using steady-state free precession sequences (SSFP), which provide diffusion weighting at relatively short echo times [133]. For articular cartilage, a three-dimensional steady-state diffusion technique, called PSIF, has been used (reversed FISP (fast imaging by steady-state precession)) [131, 132].

A variant of DWI is diffusion tensor imaging (DTI), which enables the measurement of diffusion anisotropy. In this technique, diffusion gradients are applied in at least six orientations and the data is fitted to a diffusion tensor model. Based on the determined diffusion tensor, the localized orientation of preferential diffusion and the localized diffusion anisotropy can be determined [33, 127, 130, 134]. DTI correlated with the orientation of collagen fibrils, with collagen disruption and cartilage degeneration [33, 135, 136]. However, because cartilage is characterized by a relatively low diffusion anisotropy, a reliable determination of its anisotropy requires a high SNR on the acquired diffusion data [2, 127, 137, 138].

#### 3.1.5. Segmentation of Cartilage after Cartilage Repair Procedures

Segmentation is required to obtain quantitative values for each cartilage region of interest (ROI) from the acquired images. Most of the segmentation is so far done either manually or semiautomatically. For MR imaging at the knee, sagittal or coronal planes are obtained. Kurkijarvi et al. used both planes and found slight differences between the quantitative values [125]. Segmentation may either be performed directly on the quantitative source images or on SPGR or DESS images with higher spatial resolution, which can be superimposed in the following. For cartilage repair analyses, most studies segmented the cartilage repair area, plus morphologically normal appearing cartilage of the same compartment or other compartments. Analyses were performed by either correlating absolute values from the repair tissue or by using an index, utilizing the ratio of repair tissue over normal cartilage [139, 140].

Normal hyaline cartilage has a zonal stratification, which can be detected using maps of quantitative MRI biomarkers combined with laminar or zonal analyses [34, 35, 141–145]. Based on the known anatomical ultrastructure, cartilage may be divided into three or four zones. Few studies performed laminar analysis by dividing the entire cartilage in three layers [74]. However, spatial resolution is limited in the quantitative sequences. Therefore, most studies analyzed a superficial and a deep layer [140], reflecting the hyaline cartilage ultrastructure and thus showing, for example, a significant increase of T2 relaxation times from the deep to the superficial cartilage layer [144, 145].

### 3.2. Findings for Quantitative Imaging of Cartilage Repair

Different cartilage repair techniques are known to result in repair tissues with different histological compositions that vary during maturation [14]. Hyaline-like cartilage with a normal amount of PG was described to have a better clinical outcome and less therapy failure than fibrous cartilage [146]. After Mfx, tissue has been mostly reported as fibrous cartilage [40, 41]. After MACI, tissue has been characterized as hyaline-like [42]. Osteochondral transplants consist of about 80% of hyaline cartilage and of about 20% of fibrous cartilage [43, 147–149]. Further, Mfx repair tissue was described to consist disorganized cartilage and a reduced PG content, while MACI repair had a normal zonal collagen organization [150–153]. Results from quantitative imaging also suggest a difference in composition and structure between the repair tissues after various repair techniques [39].

#### 3.2.1. Cartilage Repair Findings for T2 Relaxation Time Measurements

**Figure 1:**
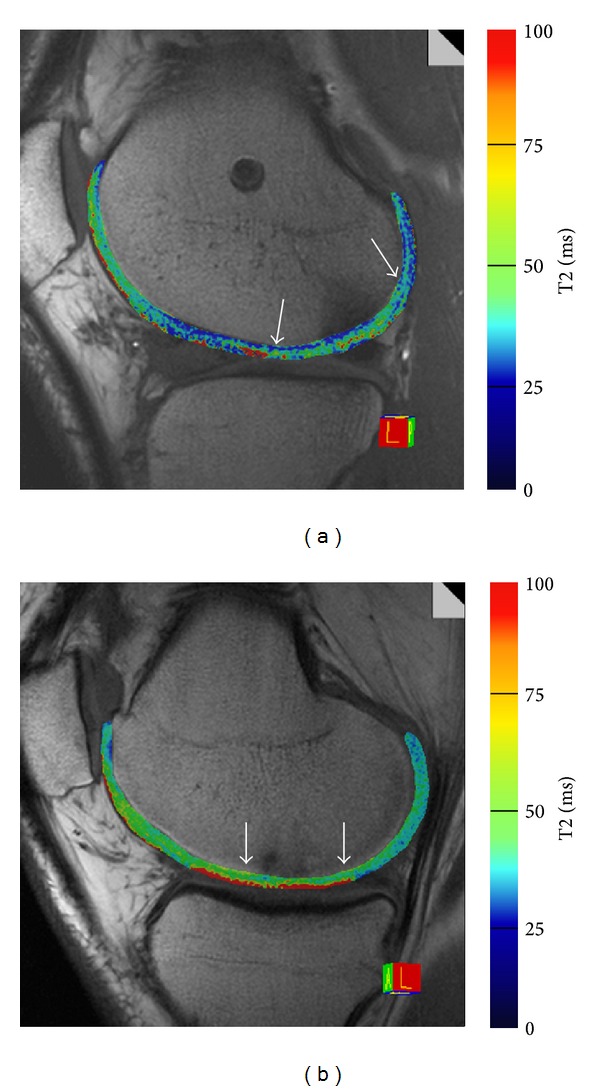
Left figure: T2-MACI. Quantitative T2 mapping of a 39-year-old male patient 24 months after MACI of the lateral femoral condyle (marked by arrows). The cartilage repair tissue showed a comparable ultrastructure compared to the surrounding native cartilage. Right figure: T2-MFX. Quantitative T2 map of a 31-year-old male patient 6 months after microfracture therapy of the medial femoral condyle (marked by arrows). The cartilage repair tissue showed still clearly increased T2 values. T2 maps were reconstructed using a multiecho spin echo (SE) acquisition, with a TR of 1200 ms and 6 TEs of 13.8, 27.6, 41.4, 55.2, 69, and 82 ms. The field of view was 160 × 160 mm, the pixel matrix was 384 × 384, and the voxel size was 0.4 × 0.4 × 3.0 mm. The bandwidth was 228 Hz/pixel, with 12 slices; total acquisition time was 4:09 minutes.

T2 relaxation time measurements seem to be promising for determining different structural tissue characteristics and to monitor the maturation process [14, 50, 69, 132, 140, 154]. It was demonstrated that fibrous cartilage repair tissue showed lower T2 relaxation times than normal hyaline cartilage [39]. Correspondingly, lower T2 values were observed for repair tissue after Mfx as compared to MACI repair tissue, while no differences in Lysholm or MOCART scores were detected (Figure 1) [132, 144, 155]. T2 values differed between MACI at different knee compartments, for different MACI scaffolds and different Mfx techniques [156–158]. One additional realization of biochemical MR imaging may be an “unloading” approach, acquiring one T2 map directly at the beginning and another T2 map at the end of the MR protocol [74, 75, 159].

Holtzman et al. reported that 3 to 6 months and 1 year after OAT T2 values showed no significant difference between repair cartilage and normal cartilage [139]. Salzmann et al. found that T2 values were higher in repair tissue after OAT as compared to normal cartilage and as compared to MACI repair tissue 3.5 years after surgery [160]. Krusche-Mandl et al. also found a significant difference of T2 values between repair cartilage after OAT and normal cartilage 8 years after surgery [26].

Maturation processes were also described for Mfx. Oneto et al. found initially elevated T2 values for Mfx repair tissue, which approached values of normal cartilage after two years in case of graft integrity and lower T2 values for therapy failure [161]. Theologis et al. observed that at 3 to 6 months after surgery Mfx repair tissue had significantly higher T1rho and T2 values relative to normal cartilage [140]. At 1 year, T2 values of repair tissue decreased to values comparable to normal cartilage (T1rho remained significantly different). In two different studies, about 2.3 and 2.7 years after surgery, Welsch et al. detected reduced T2 in cartilage repair areas after Mfx, whereas, after MACI, T2 was similar to normal cartilage [132, 144]. The laminar analysis showed the existence of a zonal stratification at 3–6 months after Mfx, which was lost after 12 months (but persisted for T1rho) [140]. Most studies however reported that normal zonal variation was lacking after Mfx [39, 83, 142, 144, 162].

Tissue maturation processes can also be depicted after ACI [39]. Kurkijarvi et al. showed that T2 values for ACI repair tissue were higher and more heterogeneous than T2 of normal control cartilage about one year after surgery with a lack of zonal organization [125]. T2 relaxation times were higher for repair tissue than for normal cartilage 3–13 months after ACI, but no significant difference was detected at later time points in several studies (after 19–42, 12–59, and 20 months) [144, 145, 162, 163]. However, T2 of MACI repair tissue generally decreased during longer postoperative intervals [144, 162, 164]. According to Salzmann et al., repair tissue had significantly lower T2 values than normal cartilage about 3.5 years after MACI [160]. Zonal differences between the cartilage layers have been reported previously [39, 62, 144, 165]. However, these zonal differences seem to vary during the maturation process after ACI. After one year, no zonal stratification was detected [125]. At later time points during the second year after MACI T2 zonal organization approached that of control cartilage and persisted during longer followups, as opposed to no zonal variation after Mfx [141, 144, 145, 162, 163]. Overall, these findings for T2 relaxation time measurements of cartilage repair tissue may correspond to the described histological differences of the tissue and indicate a maturation of both layers with a decrease in water content and an increase of collagen content and orientation [132].

**Figure 2:**
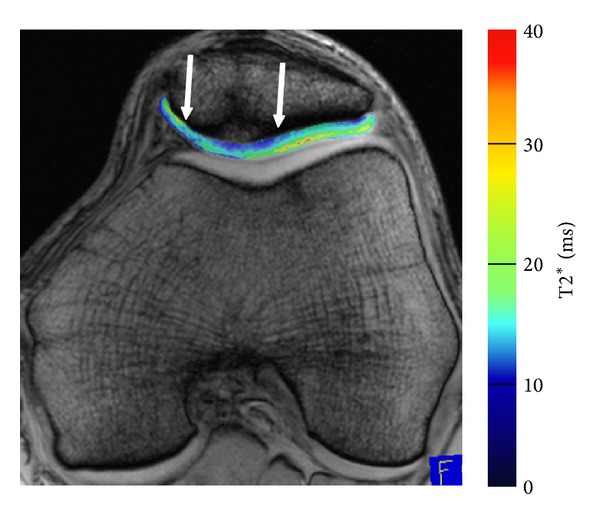
T2*-MFX. Quantitative T2* imaging of a 42-year-old male patient 24 months after microfracture therapy of the patella cartilage (marked by arrows). The cartilage repair tissue showed reduced T2* values (ms) and in comparison to the adjacent native cartilage less zonal organization. T2* maps were reconstructed using a multiecho gradient echo (GRE) acquisition, with a TR of 600 ms and 6 TEs of 4.2, 11.3, 18.5, 25.6, 32,7, and 39.9 ms. The field of view was 160 × 160 mm, the pixel matrix was 384 × 384, and the voxel size was 0.4 × 0.4 × 3.0 mm. The bandwidth was 260 Hz/pixel, with 12 slices; total acquisition time was 2:35 minutes.

Studies reported that T2* mapping may provide additional information on cartilage repair tissue (Figure 2) [39]. Stelzeneder et al. reported that at 1-year followup after arthroscopic autologous collagen-induced chondrogenesis (ACIC) T2* was not significantly different between repair tissue and normal cartilage [166]. Using a GRE T2* approach with the same T2* sequence, Welsch et al. found 2.3 years after MACI a comparable T2 between repair tissue and normal cartilage but a lower T2* in repair tissue. Zonal stratification was also more pronounced for T2* than for T2 [84]. This might be due to the influence of local susceptibility variations on T2*, at the macroscopic level, that is, associated with the bone/cartilage interface or at the microscopic level, that is, associated with the underlying microstructure of the cartilage. For Mfx, comparable to MSME T2 mapping, no spatial variation was observed for cartilage repair tissue after Mfx using T2* mapping [83]. At 7 T, T2 and T2* mapping was applicable, but with overall lower relaxation times and a reduced zonal (laminar) appearance of articular cartilage [72, 167].

At the talus, ACI repair tissue showed normal T2 values in a 5-year and in a 10-year followup [168, 169]. Repair tissue showed comparable T2 values to hyaline cartilage 2 years and 4 years after bone marrow derived cell transplantation at the talus [170, 171]. T2 differed significantly between the superficial and deep layers 9.5 years after Mfx, detected via 7 T MRI [141].

#### 3.2.2. Cartilage Repair Findings for T1rho Relaxation Time Measurements

**Figure 3:**
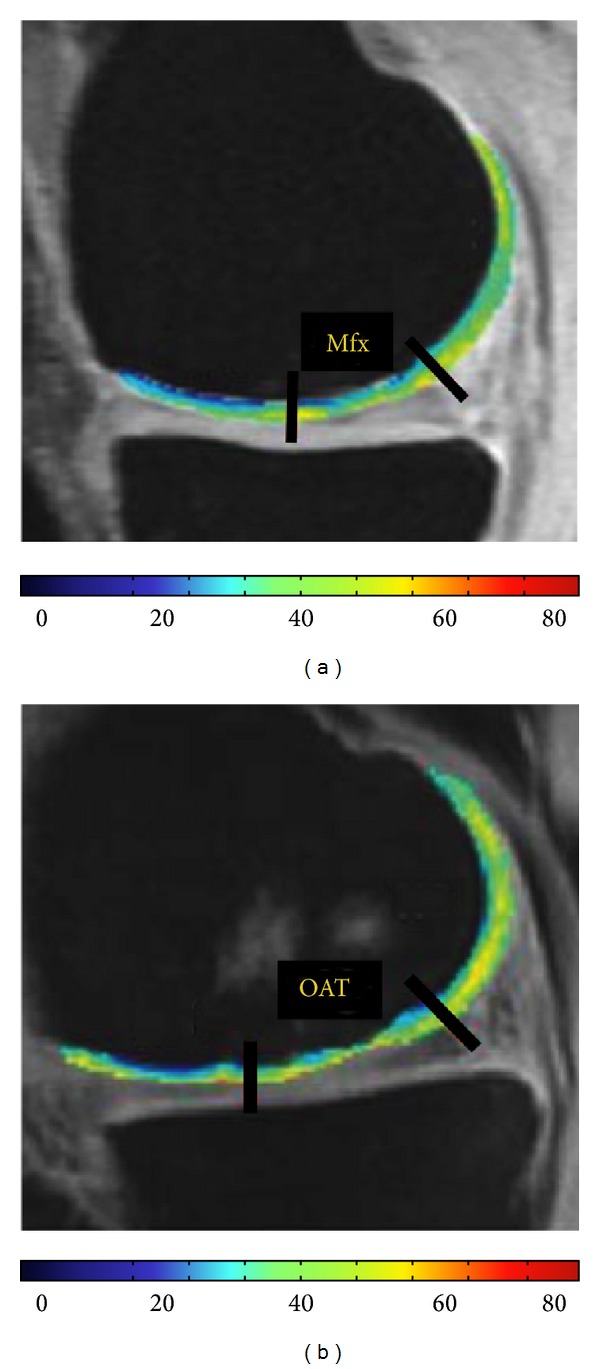
Left figure: T1rho-microfracture (Mfx). Quantitative T1rho mapping at the knee 1 year after Mfx of the medial femoral condyle. At the 1 year followup, the cartilage repair tissue still showed elevated T1 rho values as compared to surrounding cartilage. Right figure: T1rho-OAT. Quantitative T1rho map after OAT therapy of the medial femoral condyle. The cartilage repair tissue showed slightly higher T1rho values than normal surrounding cartilage. T1rho images were acquired using spin-lock techniques and 3D SPGR acquisition. TR was 9.3 ms. TE was 3.7 ms. The field of view was 140 × 140 mm. The pixel matrix was 256 × 192. The slice thickness was 3 mm. The bandwidth was 31.25 kHz. The views per segment (VPS) were 48. The time of recovery (Trec) was 1.5 s. The time of spin lock (TSL) was 0, 10, 40, and 80 ms and the spin-lock frequency (FSL) was 500 Hz.

First efforts have been made to apply T1rho relaxation times for assessment of cartilage repair tissue [139, 140, 172]. A differentiation between different tissue types after cartilage repair procedures was possible by applying T1rho mapping, in particular, in combination with T2 mapping [139]. To date, T1rho has been applied to imaging of repair tissue after Mfx and OAT (Figure 3).

Holtzman et al. reported that cartilage repair tissue after OAT showed slightly higher T1rho values than normal surrounding cartilage at 3 to 6 months and at 1 year after surgery [139]. On the contrary, T2 values were not significantly different between repair cartilage and normal cartilage. There was no difference found for T1rho and T2 values between the time points 3 to 6 months and 1 year [139].

After Mfx, Holtzman et al. observed significantly higher T1rho (and T2) values for cartilage repair tissue as compared to surrounding cartilage tissue 3 to 6 months after surgery [139]. Also Theologis et al. reported that T1rho values of cartilage repair tissue after Mfx remained higher than normal cartilage after 1 year as opposed to T2 values [140]. Laminar analysis showed significantly higher T1rho for both, the deep and the superficial layer 3–6 months after Mfx [139]. At 1-year followup, T1rho values of the deep layer decreased, indicating a maturation regarding PG content [140].

#### 3.2.3. Cartilage Repair Findings for dGEMRIC Measurements

**Figure 4:**
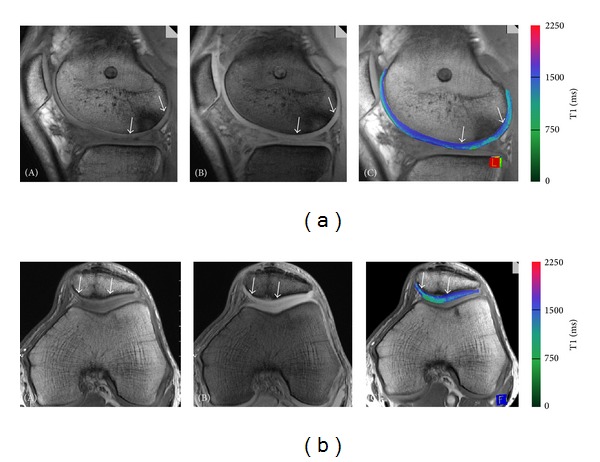
Upper row: dGEMRIC-MACI. Delayed gadolinium-enhanced MRI of cartilage (dGEMRIC) of a 39-year-old male patient 24 months after MACI of the lateral femoral condyle (marked by arrows). Images (A) and (B) represent the raw images with the different flip angles on which the dGEMRIC-T1 map is based (C). The T1-dGEMRIC map shows slightly reduced GAG content in the repair tissue. Lower row: dGEMRIC-MFX. Delayed gadolinium-enhanced MRI of cartilage (dGEMRIC) of a 42-year-old male patient 24 months after microfracture therapy of the patella cartilage (marked by arrows). Images (A) and (B) represent the raw images with the different flip angles on which the dGEMRIC-T1 map is based (C). The T1-dGEMRIC map showed a clearly reduced GAG content in the repair tissue. For T1-Gd mapping, we used a 3D gradient echo (GRE) sequence with a TR of 15 ms, a TE of 1.95 ms, and two flip angles of 5° and 18.6°. The field of view was 160 × 160 mm, the pixel matrix was 384 × 384, and the voxel size was 0.4 × 0.4 × 3.0 mm; the bandwidth was 480 Hz/pixel and 22 slices were assessed with a total acquisition time of 3:40 minutes.

dGEMRIC measurements correlate with cartilage degeneration and may predict the development of OA due to their sensitivity for GAG content of hyaline cartilage [20, 21]. dGEMRIC is increasingly applied to assess cartilage repair tissue. Watanabe et al. reported that dGEMRIC measurements correlated with GAG content of ACI grafts [119]. Few studies analyzed OAT and Mfx using dGEMRIC. T1-Gd values of OAT were comparable to normal cartilage, about 1 and 2 years after surgery with a normal zonal stratification [126, 173]. Significantly different relative delta relaxation times were found for Mfx and MACI, suggesting a lower PG content of the repair tissue after Mfx as compared to MACI (Figure 4) [165].

There are more studies analyzing repair tissue after ACI [39, 63, 69, 124, 125, 132, 165, 174–176]. Higher delta relaxation rates and lower T1-Gd values, indicating lower GAG contents, were reported for repair tissue than for normal cartilage at baseline and 1 year, 3–13 months, and 9–42 months after MACI [35, 102]. On the other hand, T1-Gd values were comparable to normal cartilage 10–15 months and 2–24 months after surgery. Several studies found significant differences of dGEMRIC values between <1 year of followup and later time points after MACI, correlating with improving GAG contents [106, 110, 124]. However, Trattnig et al. reported that T1-Gd was lower in repair tissue than in normal cartilage 2.8 and 4.7 years after MACI [23, 165]. About 4 and 5 years after MACI, a stable T1-Gd was observed by Brix et al. [175]. T1-Gd was not significantly different between repair tissue and normal cartilage 9 to 18 years after ACI according to Vasiliadis et al. [177]. Laminar analysis of dGEMRIC measurements did not show a significant difference between superficial or deep tissue as compared to the control cartilage 1 year after ACI [125]. Pinker et al. detected a tendency toward an increase in global and zonal GAG content in the deep zone of the transplant after 1 year [35].

dGEMRIC was also applied for postoperative assessment after MACI at the ankle. Domayer et al. reported no significant difference between the delta relaxation rates in repair tissue and normal cartilage [117]. Wiewiorski et al. found significantly higher delta T1 values for MACI at the talus than for normal cartilage [52].

#### 3.2.4. Cartilage Repair Findings for Diffusion Imaging

**Figure 5:**
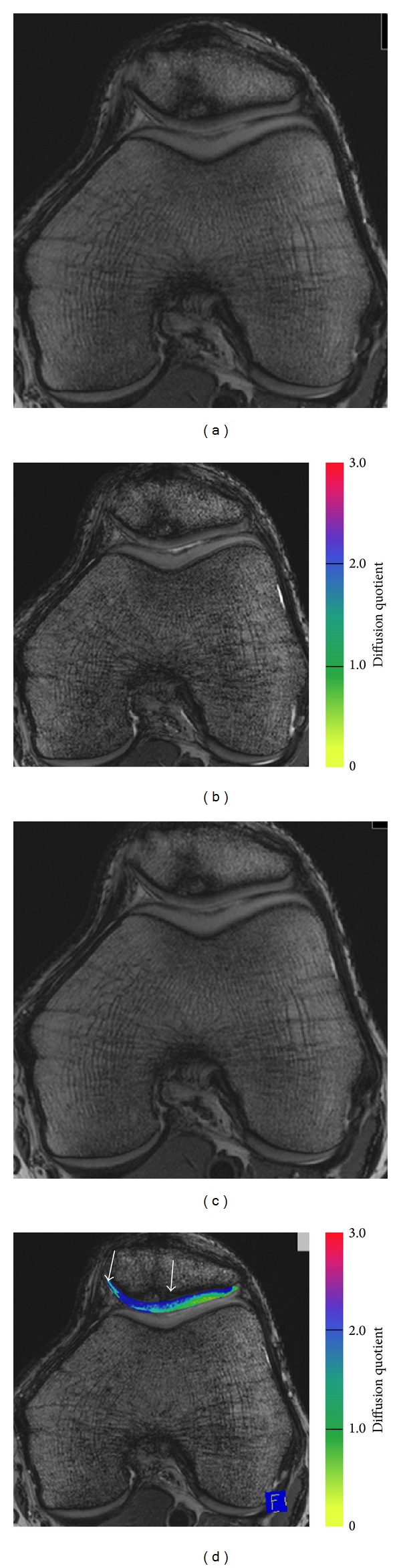
Diffusion weighted imaging (DWI) of a 42-year-old male patient 24 months after microfracture therapy of the patella cartilage (marked by arrows). Images (a) and (b) represent the raw images of two different diffusion directions, whereas image (c) is the reversed FISP sequence without any diffusion quotient. As visualized in the diffusion map (d), the diffusivity was clearly increased in the cartilage repair tissue compared to the surrounding cartilage. DWI maps were reconstructed using a three-dimensional, balanced, steady-state gradient echo pulse sequence with diffusion weighting (3D DWPSIF) (reversed FISP = fast imaging with steady-state precession), with a TR of 16.3 ms and TEs of 6.1 ms. The field of view was 160 × 160 mm, the pixel matrix was 384 × 384, and the voxel size was 0.4 × 0.4 × 3.0 mm, with 3 slices (through the area of cartilage repair); total acquisition time was 6:48 minutes.

Diffusion imaging may provide additional information for evaluation of cartilage repair tissue (Figure 5) [32, 62, 131, 132, 178]. DWI distinguished healthy cartilage from cartilage repair tissue in Mfx and MACI 2.7 years after surgery [132]. Mamisch et al. reported that diffusion in repair cartilage after MACI was significantly higher than in normal cartilage. There was a decrease between the earlier and the later time point after surgery [178]. Also, Friedrich et al. [131] found that after MACI, in a group with the first exam less than 13 months after surgery and a 1-year follow-up exam, diffusion quotients were significantly lower at the followup. In a group with the first exam more than 13 months after MACI and a 1-year follow-up exam no statistically significant differences in diffusivity were found between the two time points. This may reflect cartilage maturation [131].

After MACI at the ankle joint cartilage repair tissue showed no significant difference in T2 and T2* relaxation times compared to the control cartilage, but it showed a higher diffusivity [179]. Apprich et al. examined cartilage repair at the ankle 5 years after Mfx and 4 years after MACI using DWI [180]. Diffusivity was similar between normal cartilage and MACI; however, Mfx repair tissue showed significantly different DWI values compared to both, MACI and normal cartilage [180].

#### 3.2.5. Correlations of Different Biomarkers

Since GAG depletion generally occurs before collagen disruption, cartilage imaging techniques with sensitivity for different pathological mechanisms may detect not only different stages of cartilage degeneration but also different stages of cartilage repair [85, 181]. Further, different techniques may have different dynamic ranges such as T1rho and T2 [181] or dGEMRIC and T2 [107]. Therefore, it seems likely that, besides morphological imaging and clinical scores, a multimodal approach combining different quantitative MR imaging techniques may enable a more comprehensive characterization of cartilage repair [125]. A multimodal approach was already performed by several study groups that analyzed cartilage repair [72, 83, 107, 125, 132, 139, 140, 179]. Nevertheless, more cross-validation of different MR imaging methods needs to be performed in future studies. This is especially important to gain more information on the use, the meaning, and the significance of the different techniques in assessing cartilage and cartilage repair tissue.

#### 3.2.6. Correlation with Morphological and Clinical Scores

Several studies have evaluated the correlation of MR cartilage biomarkers with clinical scores. For the majority of quantitative MRI parameters, limited or no correlation was reported. A correlation between Lysholm scores and T2 was found by Welsch et al., Krusche-Mandl et al., and Domeyer et al. [26, 132, 155]. Giannini et al. found a correlation of T2 maps with clinical results after bone marrow derived cell transplantation at the talus [171]. Welsch et al. found a correlation of Lysholm scores and DWI [132]. Bekkers et al. found a similar improvement of clinical scores and dGEMRIC measurements from baseline to a 12-month followup [106]. Two systematic meta-analyses were recently published analyzing the correlation of MRI parameters with clinical outcome [37, 46]. A correlation between clinical outcome and MOCART or Henderson score was found in only nine studies (28%) [37, 46]. The authors reported a weak to moderate correlation between clinical outcome and T2 [37]. Overall, de Wind et al. stated that strong evidence to determine whether morphological MRI is reliable in predicting clinical outcome after cartilage repair is lacking [37].

Regarding morphological MR scores, significant correlations were found between MOCART score and DWI after Mfx and MACI [132, 180]. Most authors however stated that quantitative MR imaging did not correlate significantly with morphological MR scores [132, 178].

## 4. Discussion

Based on findings in early cartilage degeneration, focal cartilage defects, and OA, quantitative MR imaging may be helpful for assessing biochemical composition and maturation of cartilage repair tissue, its ultrastructural outcome and intraindividual quality. To date, most studies involved ACI, although studies investigated OAT and Mfx. For all biomarkers, studies reported that a difference of quantitative values for repair tissue after Mfx compared with ACI or OAT could be detected. Most studies were performed at the knee; few studies were performed at the ankle. The latter showed more inconsistent results that additionally differed from those at the knee.

At the knee, T2 relaxation time measurements, which are sensitive to collagen disruption, seem to be the most established quantitative imaging techniques so far. Summarizing the findings of the different studies, T2 values for OAT were initially similar to normal cartilage but increased years after surgery. For Mfx, initially elevated T2 values were found that decreased to values similar to normal cartilage about 1 to 2 years after surgery and decreased further during longer followup. Mfx did not show a zonal stratification. Mfx generally showed lower T2 values than ACI. ACI also presented elevated T2 values initially that decreased to normal values during the second year after surgery. However, also after ACI, a further decrease of T2 values during longer followup of up to 5 years after surgery was observed. Zonal stratification appeared during the second year after surgery and persisted during longer followup. T2 mapping was frequently used in combination with PG sensitive techniques such as dGEMRIC or T1rho, thus providing complementary information. This underlines the importance of combining several quantitative MR imaging techniques, alongside with morphological and clinical evaluation for postoperative followup and monitoring after cartilage repair procedures for most conclusive tissue assessment.

T1rho was found to be elevated in cartilage repair tissue after OAT and Mfx as compared to normal cartilage up to 1 year after surgery. No longer followup was performed so far. For T1rho mapping, high SL fields are desirable, since it correlates positively with T1rho relaxation times. However, due to long durations of the SL pulse, some groups reported high SAR as a major issue for clinical application and therefore suggested limited clinical use of T1rho [7, 120, 182]. This SAR issue seems even more important at 3 T as compared to 1.5 T. However, other groups could optimize these sequences while staying within the prescribed SAR limits. Further, it was reported that T1rho was not entirely GAG specific [94].

Regarding dGEMRIC, results of current studies are more heterogeneous than for T2. For Mfx, less PG content was detected by dGEMRIC than for ACI. OAT showed normal PG content and a normal zonal stratification. Most studies were performed on ACI. In some studies, PG content, detected by dGEMRIC, was lower in repair tissue 1–3 and about 3 and 5 years after ACI. Other studies reported values comparable to normal cartilage during not only the first 2 years but also 9–18 years after surgery, as well as stable PG contents between 4 and 5 years after ACI. These diverging results may have different explanations, such as different surgical techniques, different imaging protocols, different coils, different time points of contrast agent administration, different analysis methods, and different patient cohorts. The main disadvantage of dGEMRIC is the relatively long examination time and the requirement of exogenous gadolinium-based contrast agent injection with its rare but possible side effects of nephrogenic systemic fibrosis and allergic reactions. Therefore, it has to be stated clearly that in future approaches comparable quantitative analyses may be possible without contrast injections. Further, a static state with complete equilibration after contrast agent injection cannot be reached in vivo and the GAG content of the deep cartilage layer may be overestimated [183–185]. Further, dGEMRIC may not be specific to GAG. No study has evaluated the dependency of dGEMRIC values from the intra-articular contrast concentration so far.

For DWI, the current studies mainly describe a higher diffusivity for cartilage repair tissue after MACI as compared to normal cartilage with a decrease over time. After about 2 years, DWI values remained stable. Using current routine techniques, it seems difficult to obtain precise measures of the diffusion values in cartilage, due to image distortions, limitations in contrast, and sensitivity to motion artifacts [131]. Ongoing approaches on more stable and clinical feasible sequences (e.g., SSFP approach) try to enable for quantitative DWI in cartilage and cartilage repair. Clinical application of DTI is challenging due to long acquisition times, high field strengths, and intensive data analysis.

Currently, clinical correlations are limited and clinical importance including prediction of procedure outcome needs to be demonstrated in larger cross-sectional and longitudinal cohort studies. Standards of reference have to be established in dependence on follow-up time points in long-term followup after different cartilage repair procedures. Quantitative MR imaging did rarely correlate significantly with morphological MR scores and may possibly provide supplemental information for evaluation of cartilage transplant maturation [132, 178].

To date, all quantitative imaging techniques have several technical and methodological limitations and therefore remain work-in-progress. For all sequences, manual segmentation is required which is limited by inter- and intraobserver reliability errors. To date, the difficult and time-consuming segmentation process may be one of the most important issues, which limits the broad clinical use of quantitative MR techniques. No entirely automated segmentation tool is available so far. Driven by different companies and working groups, this problem may be solved in the future, aiming for the immediate availability of quantitative values after MR acquisition.

Frequently, the surrounding cartilage has been considered as normal cartilage and was used as a reference standard for calculation of an index; however, the adjacent cartilage cannot in general be considered as healthy [39, 124, 186, 187]. Surrounding cartilage showed further deterioration after cartilage repair. This may be one explanation for the lack of correlation between morphological and biochemical MRI and clinical score. For this reason, there are upcoming approaches that consider the entire joint cartilage after cartilage repair. Looking only at the repair tissue area itself may not be representative for the entire joint. [188]. A general standard of reference for each MR biomarker is still lacking and difficult to obtain, since different acquisition methods, calibration procedures, coils, and MR scanners lead to varying results. Further, only in case of OAT, it is known that the transplant initially consists of hyaline cartilage. For ACI and Mfx, quantitative values may detect any tissue that accidentally shows quantitative values comparable to hyaline cartilage. “Normal” tissue multimodal characteristics would need to be defined for each specific tissue. It appears doubtful that quantitative imaging characteristics that were obtained in hyaline cartilage can directly be transferred to repair tissue with an unpredictable histological composition. Characterization of cartilage repair tissue by using only one imaging parameter seems unlikely. One important step would be to combine different biomarkers in a multimodal approach to gain the most conclusive information on the tissue characteristics. At the current time point, it seems challenging that all quantitative imaging techniques may be performed at once due to the long acquisition times.

## 5. Conclusions

In conclusion, noninvasive quantitative MR imaging of cartilage repair tissue has shown varying results. Multiple imaging biomarkers such as T2 mapping, T1rho, and dGEMRIC demonstrated sensitivity to biochemical alterations of cartilage repair tissue. However, results that were obtained in hyaline cartilage do not seem to be easily transmittable to repair tissue. While quantitative MR biomarkers are established for OA for cartilage repair purposes, all techniques remain work-in-progress. Acquiring accurate and clinically valuable quantitative data has proven challenging. Considering the limitations, using multimodal approaches including multiple quantitative MR biomarkers, morphological MRI, and clinical scores in combination would be most promising for clinical applications in the future.
